# Supplementary material for: An Experimental Approach to Address the Functional Relationship between Antioxidant Enzymes and Mitochondrial Respiratory Complexes
Source: Methods Protoc. 2023 Mar 24;6(2):32. doi: 10.3390/mps6020032 (PMC10142429; doi:10.3390/mps6020032)
Supplement: Supplementary file 1 [file mps-06-00032-s001.zip › mps-2269453-SI.pdf]

An Experimental Approach to Address the Functional Relationship between Antioxidant Enzymes and Mitochondrial Respiratory Complexes

Daniela Mendes [1], Ana Maria Silva [2], Maria Manuel Oliveira [3], Paula B. Andrade [1] and Romeu A. Videira [1,*]

[1] REQUIMTE/LAQV, Laboratory of Pharmacognosy, Department of Chemistry, Faculty of Pharmacy, University of Porto, Rua de Jorge Viterbo Ferreira, nº 228, 4050-313 Porto, Portugal
[2] Department of Life Sciences, University of Coimbra, Calçada Martim de Freitas, 3000-456 Coimbra, Portugal
[3] Chemistry Center—Vila Real (CQ-VR), Chemistry Department, School of Life and Environmental Sciences, University of Trás-os-Montes e Alto Douro, UTAD, 5001-801 Vila Real, Portugal
* Correspondence: rvideira@ff.up.pt

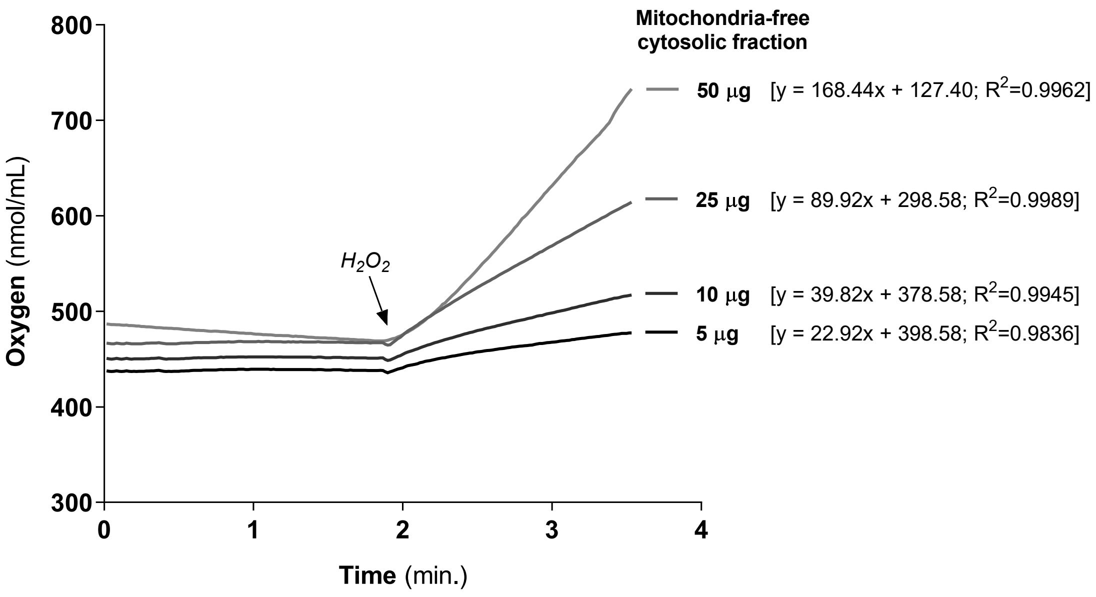

Figure S1 – Oxygen production in nmol/mL for different concentrations of mitochondria-free cytosolic fraction (5, 10, 25 and 50µg) with the same H_2O_2 concentration.
